# Prevalence and incidence of balance disorders in community-dwelling older adults: Protocol for the EPIBAS epidemiological balance study

**DOI:** 10.1371/journal.pone.0346698

**Published:** 2026-06-05

**Authors:** Pilar Montero-Alía, Antonia Fornes-Reynes, M. Carmen Rodríguez-Pérez, Rosalía Dacosta-Aguayo, Ramón Miralles-Basseda, Tamara Jiménez-Pascua, Jofre Pascual-Bielsa, M. José Durá Mata, M. Mercedes Molleda-Marzo, Lucía Ramos-García, Víctor M. López-Linfante, Maria Doladé-Botías, Marta Álvarez-Álvarez, Mercè Jiménez-González, Juan José Montero-Alía, Álvaro Suárez-Hervella, Galadriel Diez-Fadrique, Mireia Moix-Juan Torres, Leila De Gea-Espinosa, Noemí Lamonja-Vicente, Laia Mas-Pons, Claudia León-Prieto, Amaia Calderón-Larrañaga, Josep Maria Manresa-Domínguez, Pere Torán-Monserrat

**Affiliations:** 1 Unitat de Suport a la Recerca Metropolitana Nord (USR Metro-Nord), Institut Universitari d’Investigació en Atenció Primària Jordi Gol i Gurina (IDIAP Jordi Gol), Mataró, Barcelona, Spain; 2 Grup de Recerca en Indicadors i Determinants Associats a Envelliment Saludable (GRIDAES), IDIAP Jordi Gol, USR Metro-Nord, Mataró, Barcelona, Spain; 3 Centre d’Atenció Primària La Riera, Institut Català de la Salut, Mataró, Barcelona, Spain; 4 Centre d’Atenció Primària, Martorelles-Sant Fost, Insitut Català de la Salut, Barcelona, Spain; 5 Faculty of Health Sciences, International University of La Rioja, UNIR, Logroño, Spain; 6 Department of Geriatrics, Hospital Universitari Germans Trias i Pujol, Badalona, Spain; 7 Direcció Clínica Territorial de Cronicitat Metropolitana Nord, Institut Català de la Salut, Barcelona, Catalonia, Spain; 8 Centre d’Atenció Primària Ronda Prim, Institut Català de la Salut, Mataró, Barcelona, Spain; 9 Department of Physical Medicine and Rehabilitation, Hospital Universitari Germans Trias i Pujol, Badalona, Barcelona, Spain; 10 Varpa Group, Biomedical Research Institute A Coruña (INIBIC), University of A Coruña, A Coruña, Spain; 11 Department of Computer Science and Information Technologies, University of A Coruña, A Coruña, Spain; 12 Clinical and Biochemical Analysis Division, Laboratori Clinic Metropolitana Nord (LCMN), Hospital Universitari Germans Trias i Pujol, Badalona, Spain; 13 Centre d’Atenció Primària Rocafonda, Institut Català de la Salut, Mataró, Barcelona, Spain; 14 Centre d’Atenció Primària Gatassa, Institut Català de la Salut, Mataró, Barcelona, Spain; 15 Aging Research Center, NVS Department, Karolinska Institutet, Stockholm University, Stockholm, Sweden; 16 Department of Medicine, Universitat de Girona, Girona, Spain; Universita Politecnica delle Marche Facolta di Ingegneria, ITALY

## Abstract

**Background:**

Poor balance leads to falls, which can cause a loss of personal autonomy and reduced quality of life. Epidemiological studies of balance disorders (BDs) are limited. Identifying their prevalence and accessible diagnostic markers is crucial for prevention and monitoring.

**Aims:**

1) Assess the prevalence of BDs in men and women aged 65–72 using posturography and different balance tests, and track the annual incidence of BD; 2) Analyze the association between retinal microvasculature abnormalities and BD; 3) Evaluate the effectiveness of the Wii Balance Board™ for balance assessments compared to posturography; 4) Explore whether knee extensor strength and a combined measure of physical and cognitive function can serve as predictors for BD and fall risk: and 5) Conduct an external validation of the Health Assessment Tool (HAT) in the Spanish population.

**Methods:**

Two-phase, observationals study. Phase I: Descriptive, cross-sectional. Phase II: Follow-up of cohorts at 18 months. Sample: 1,316 people aged 65–75 residing in Mataró (Barcelona, Spain) shall be included in the study, excluding individuals who cannot walk independently. They shall undergo posturography, retinal photography, knee extensor strength assessment, as well as the Tinetti Test, Timed Up and Go test, Unipedal stance test, Short Physical Performance Battery (SPPB), and balance assessment using the Nintendo Wii at baseline and at the end of the follow-up period. Bimonthly phone calls shall be made to detect the occurrence of falls.

**Applicability and relevance:**

BDs are underdiagnosed and under-treated, and current awareness only scratches the surface. Expanding knowledge in this field is essential to avoid the loss of functional autonomy in individuals. Doing so will make it possible to develop specific preventive measures for the population at greatest risk of falls, thus making the approach to this significant geriatric syndrome more efficient.

The EPIBAS study is registered on Clinicaltrials.gov under the code NCT06965660.

## Introduction

The aging process is accompanied by a decline in several body functions involved in maintaining balance. Poor balance and gait disorders lead to falls, resulting in a loss of personal independence, reduced quality of life [1], stress, isolation, and decreased physical activity [2]. Injuries from falls, defined as unintentional loss of balance leading to failure of postural stability, can result in extended use of healthcare services and high healthcare costs [3,4]. There is a pressing need to anticipate falls and understand the prevalence of balance disorders (BDs) to offer rehabilitation treatments or preventive strategies for those at most risk.

Previous studies indicate that balance, as an independent factor, is associated with survival, the better the balance, the longer the survival [5,6]. Impaired proprioception, visual loss, peripheral polyneuropathy, strokes, loss of muscular strength, and vascular diseases are common causes of gait and BD in the elderly [3,7,8]. Instability in older adults may be related to the specific impairments in these systems or cumulative loss of function associated with age. This multifactorial etiology, with diverse treatments, makes it difficult to determine and address the underlying cause.

Epidemiological studies on BS are limited [9,10] and often rely on patient series to estimate prevalence. One of the most recent studies, conducted in Turkey [11], used the Berg Balance Scale and reported that 34% of elderly participants experienced BDs. Another study from the USA based its findings on self-reported survey data [12]. Literature reports a prevalence of dizziness of 3–7% in the general population, with instability rising to 17–30%. Prevalence is even higher between 65 and 69 years, reaching over 40% for adults aged 80–84 [13]. Only one epidemiological study in Spain has investigated BD prevalence at the population level in primary care [14], but it does not assess the prevalence of presbyequilibrium, the natural involution of the balance system due to aging, causing slight vestibular symptomatology and moderate disability [15].

In the context of the demographic transition towards a progressively aging population, it is essential to determine the prevalence and incidence of BDs and describe potential markers for easy detection and monitoring. Simple and practical tools are needed at the population level to identify individuals with BD, such as measuring retinal vasculature, an indirect marker of brain vascular disease, with non-mydriatic cameras, and using balance platforms like the Wii Balance Board™.

Some studies describe a correlation between postural instability (measured by unipedal stance time) and asymptomatic cerebral microcirculation abnormalities (lacunar stroke, periventricular hyperintensities, and microhemorrhages) detected through Magnetic Resonance Imaging (MRI) [16–19] in healthy adults, potentially representing an early biomarker for cognitive decline. The ocular fundus offers a window to observe the circulatory system. Findings in retinal microcirculation predict functional decline and slowed gait in older adults [20], reflecting the status of the cerebral vascular tree [21] and serving as biomarkers for cerebral infarcts, strokes, and white matter lesions [22].

The retina can be considered an extension of the brain; in embryological terms, the retina is an extension of the diencephalon. There is evidence of an association between retinal vessel density and cognition as measured by MoCA in healthy older adults [23]. Examining the retina with non-mydriatic cameras is a widely employed technique in primary care since it is quick, safe, and non-invasive and allows direct observation of the ocular fundus and retinal microcirculation. A study performed on a Catalan cohort allowed researchers to describe abnormalities detected in the arteriovenous (A/V) ratio in retinal microcirculation using images taken with a non-mydriatic retinal camera as potential biomarkers of silent cerebral ischemia [24]. It is feasible that cerebral microcirculation abnormalities affect the postural balance system, and there may be a relationship between postural balance markers and cerebral microcirculation markers observed in the retina. These findings underscore the importance of retinal vasculature assessment as a tool for the early detection of cerebrovascular disease.

Balance platforms are suitable for taking specialized measurements and use in postural balance rehabilitation. However, their high cost and the challenges of transportation and management make them less practical for clinical and community settings. Various studies reveal there is an acceptable correlation between the Wii Balance Board™ and force balance platforms used for Posturography, which are considered the laboratory gold standard. However, a systematic review carried out by Clark et al. identifies some limitations in the use of the Wii Balance Board™ to assess balance in clinical settings [25]. Our group performed a clinical trial to evaluate whether using the Wii Balance Board™ could improve balance, reduce falls, or improve fear of falling [26]. It yielded positive results regarding fear of falling after participants had completed a training program.

Aging affects individuals differently, leading to varying health trajectories impacting healthcare systems and individual needs. Tools that help detect the most susceptible or at-risk subjects are invaluable, and the Health Assessment Tool (HAT) appears to be one of them [27]. HAT is a simple index for assessing individuals’ clinical and functional status, allowing early detection of health changes and prediction of the care needs of older people. Developed and validated based on the Swedish National Study on Ageing and Care (SNAC), HAT presents the advantage of not having any floor or ceiling effect. It is based on five clinical indicators: physical function (walking speed), cognitive function (Mini-Mental State Examination [MMSE]), number of chronic disorders, dependence on instrumental activities of daily living (IADL), and personal activities of daily living (ADL).

Previous studies highlight the need to validate the HAT tool in other populations [28]. We aim to test HAT’s ease of use, feasibility, and predictive performance concerning BD and presbyequilibrium in a Spanish cohort of older adults. BDs are a frequent reason for consultation in primary care [14], but they do not receive the attention they deserve due to a lack of resources or knowledge on how to proceed. This study aims to provide objective data on the prevalence and incidence of this problem in our community to plan future preventive interventions.

## Study rationale and hypotheses

The aging process is accompanied by a decline in several body functions involved in maintaining balance. Poor balance and gait disorders lead to falls, resulting in a loss of personal independence and reduced quality of life. Despite the significant impact of injuries from falls and their associated healthcare costs epidemiological studies on balance disorders (BDs) are limited, and current awareness only scratches the surface. There is a pressing need for accessible diagnostic markers and simple, practical tools in primary care – such as retinal microvasculature assessment, the Wii Balance Board™, and the Health Assessment Tool (HAT) – to identify individuals at risk and provide early preventive strategies. Hence, the main hypotheses stated in this study are: 1) The presence of BD, objectively measured by posturography, is associated with an increased risk of falls and worse fall-related functional outcomes in the general population, compared to individuals without such disorders; 2) Cerebral microcirculation abnormalities and early structural alterations in the brain may impact the postural balance system. These can potentially be measured through the observation of retinal microvasculature using non-mydriatic camera images of the ocular fundus; 3) Measurements of balance using the Wii Balance Board™, knee extensor strength, retinal microvasculature abnormalities, and the comprehensive Health Assessment Tool (HAT) may predict the incidence of BD, the resulting risk of falls, and the subsequent use of Health services in adults aged 65 and over.

Providing objective data on the prevalence and incidence of this problem is essential to plan future preventive interventions. Identifying individuals at the greatest risk will allow for the development of targeted fall prevention and rehabilitation programs, ultimately helping to reduce the loss of autonomy and enhance the quality of life for the elderly population.

## Study aims

*Primary aims*

1. Determine the prevalence of BD assessed using posturography in a cohort of men and women aged 65–75, and track the annual incidence of BD at an 18-month follow-up.
2. Analyze the association between retinal microvasculature abnormalities, observed in retinal photographs, and the markers of BD detected through posturography.
3. Analyze the validity of balance assessments taken with the Wii Balance Board™ in comparison to posturography, the current gold standard method.
4. Determine whether measurements of knee extensor strength and a composite measure of physical and cognitive function can predict BDs and fall risk.

*Secondary aims*

1. Conduct an external validation of the Health Assessment Tool (HAT) in the Spanish population.

## Methods

### Study design

This study will be performed and reported according to the Strengthening the Reporting of Observational Studies in Epidemiology Criteria (STROBE) [29].

This is a two-phase, observational study:

Phase 1: A descriptive, cross-sectional study on the prevalence of BDs in individuals aged 65–72.

Phase 2: A longitudinal, population-based cohort study to determine the occurrence of postural BDs and their consequences over an 18-month follow-up period in individuals aged 65–72. The potential consequences include: a) Falls: Defined as involuntary events in which an individual loses their balance and ends up on the floor or another hard surface. b) Fall-related consequences: Including contusions, fractures, hospitalization, and death.

Based on the above-mentioned cohort data, the external validation of the HAT scale will be performed by analyzing its predictive capacity for BDs and the use of health services after 18 months of follow-up (hospital admissions, 30-day readmissions, days spent in the hospital, primary care visits, and specialist care visits).

### Setting

The study is set in primary care, targeting people aged 65–75 living in Mataró (Barcelona, Spain).

### Participants

The target population is an age-based cohort of people aged 65–75 living in Mataró. This age range was selected to find individuals who do not yet have BDs, enabling the calculation of the incidence. According to the 2017 municipal register, and the population’s age structure, there are 2,215 people in this age range. A total of 1,316 people will be randomly selected from the Primary Care Information System (SIAP) database, which includes health cardholders and covers nearly the entire population. The selection process will be conducted using a computer-generated random sequence to form a sample stratified by gender and age. Our research team will contact the selected participants using the phone numbers registered in the SIAP and invite them to participate. To ensure robustness and minimize non—response bias, a protocol of up to six call attempts on different days and at different times will be implemented for each candidate before considering them unreachable.

Eligibility criteria: 1) Aged 65–75 years at the time of recruitment. 2) Resident of Mataró (Barcelona, Spain) and registered in the Primary Care Information System (SIAP). 3) Able to walk independently, defined as the ability to walk at least 10 meters without the assistance of another person, although the use of technical aids (e.g., cane, walker) is permitted.

Exclusion criteria: 1) Permanent inability to walk or requirement of human assistance for mobility. 2) Terminal illness, as a document in the primary care electronic medical record. 3) Severe cognitive decline, as assessed by the Pfeiffer questionnaire or Mini-Mental State Examination (MMSE) during the initial evaluation, or a known diagnosis of advanced dementia. 4) Significant language barrier that prevents the completion of clinical scales or understanding of study procedures. 5) Lack of phone access for follow-up calls.

### Sample size

We expect 33% of the target population (2,215 subjects) to present a BD at recruitment. In Spain, between 14% and 46% of adults aged 65 and over experience one fall per year [30]. In the worst-case scenario, if the cohort with BDs has a fall incidence of 50%, to determine a risk ratio of 1.3 compared to the group without BDs, the latter group’s incidence would need to be around 38.5%. Accepting an alpha risk of 5% and a beta risk lower than 20%, with a dropout rate of 20% at follow-up, 329 subjects would be needed in the BD group and 987 in the non-BD group to detect a minimum risk ratio of 1.3 (objective 2). This total sample of 1,316 subjects allows for determining a prevalence of falls of 50% with ±2.7% precision (objective 1).

Regarding the retinal A/V ratio, a previous study on hypertensive patients observed a prevalence of pathological A/V ratio of 15% [21]. Given that at least 75% of the Spanish population presents with hypertension. Therefore, of the total cohort of 1,316 individuals, we expect 148 to show a pathological A/V ratio. If 33% of the population is expected to have BDs, in this sample of 148 individuals, a prevalence of 12.5% in patients with a normal A/V ratio will represent a significant difference (aim 2).

## Variables

### Functional capacity and postural balance

- *Tinetti Test: balance and gait assessment*: The Tinetti Test (TT) assesses balance (sitting and standing) with 14 items (score out of 24) and gait with 10 items (score out of 16), resulting in a total score out of 40. Higher scores indicate better performance. The test showed good interrater reliability and concurrent validity [31,32].
- *Unipedal Stance Test:* The Unipedal Stance Test (UPST) measures static balance ability [33]. It can be conducted with eyes open and closed. Participants must remain in unipedal for 10 seconds on each side, first on the dominant side and then on the non-dominant side. After 30 seconds of recovery, the test is repeated on the opposite side.
- *Time Up and Go test:* The Time Up and Go test (TUG) measures the time it takes for a subject to stand from a chair, walk three meters, turn around, and sit down again. Times begin when the subject stands up and stops when seated [34,35].
- *Balance Assessment Using the Nintendo Wii Console:* This includes the one leg balance test and center of gravity assessment, as well as Romberg Test variants: Eyes Open (REO), Eyes Closed (REC), and on Foam Pad (RuFP), as performed in the posturography test. The Nintendo Wii provides accurate measures of body center of pressure (COP), an important metric for balance stability assessment approximating the body’s center of mass. The Romberg test assesses gait disturbance caused by abnormal proprioception, disequilibrium from central vertigo, and peripheral vertigo [36]. Patients are asked to stand with feet together, arms next to the body, first with eyes open and then closed. The patient tries to maintain his balance. The test is scored by counting the seconds the patient can stand with eyes closed.
- *Posturography:* Static posturography is performed in four different situations using the Romberg test and stability limits with the NedSVE/IBV^®^ platform [37]. Postural stability is quantified using the Dinascan/IBV force platform (600 × 370 mm of active area, 100 mm in height and 25 kg in weight) with the NedSVE/IBV^®^ system. Participants find the most stable position while barefoot, with relaxed arms and feet at a 30° angle. Four static conditions are measured, each lasting 30 seconds. Conditions: Romberg test with eyes open (ROA), Romberg test with eyes closed (ROC), Romberg test with eyes open on a foam pad (RGA), and Romberg test with eyes closed on a foam pad (RGC).

Stability limits are assessed by maximum anteroposterior (AP) and mediolateral (ML) displacement (mm), representing the furthest point reached by the centers of pressure in the anteroposterior and mediolateral axes during the recording time. Posturography evaluates whether there is a BD and in which component it occurs (visual (VIS), vestibular (VES), or somatosensory (SOM) [37].

The balance assessment is based on the comparison of the parameters that best discriminate the pathology of the general population with those obtained from patterns of normality segmented by age (database of the Institute of Biomechanics of Valencia). Ratings are displayed in percentages, so that results other than 100% reflect discrepancy with respect to normal values. It is considered pathological when the results are less than 95%, indicating that these subjects are more likely to present a disorder in the studied system. Pathological values for the limits of stability are considered to be below 85% [37].

- *Short Physical Performance Battery (SPPB)* [38]*:* The Short Physical Performance Battery is an objective tool for measuring functional capacity, balance, and lower limb strength in adults over 65. The test includes three domains to assess functional mobility: gait, sit-to-stand, and balance. A score below 8 indicates mobility and physical exercise limitations and is associated with a higher risk of mobility disability and, consequently, a higher risk of falls. A score of 8 or higher is considered within the normal range*.*
- *Muscle Strength of Lower Limbs*: Maximal isometric muscle strength of knee extension following the protocol described by Gandevia [39].
- *Gait Speed:* Measured over 6 meters or 2.44 meters if the participant reports walking slowly.
- *Functional capacity* was assessed using the *Lawton-Brody IADL scale* [40]*.* This scale assesses instrumental activities of daily living (IADLs), which measure a person’s ability to live independently. It includes tasks such as managing finances, shopping, using the telephone, and handling transportation. *The Barthel ADL index* is a scale that measures basic activities of daily living (ADLs), assessing a person’s ability to perform essential self-care tasks such as bathing, dressing, toileting, transferring, and eating*.*
- *Physical activity measured using the abbreviated Spanish version of the Minnesota Leisure-Time Physical Activity Questionnaire (VREM)* [41]*:* The VREM is a short Spanish version of the Minnesota Leisure Time Physical Activity Questionnaire (CAFM). It is a tool designed to assess the quality and quantity of physical activity during leisure time. It consists of six items that evaluate physical activity carried out in the past month, considering only activities performed during leisure time.

### Health assessment tool

1. Physical function is assessed by measuring gait speed.
2. Functional cognitive status is determined using the Mini-Mental State Examination (MMSE), with a score ranging from 0 (the worst outcome) to 30 (the best).
3. Chronic morbidity is evaluated by counting the number of diagnosed chronic conditions. Diagnoses, based on the ICD-10 system, are gathered from the patient’s electronic medical record.
4. Mild disability is measured using the Lawton-Brody scale, assessing instrumental activities of daily living (IADLs) that the individual can not perform independently. Four activities will be included in the analyses: shopping, managing finances, using the telephone, and using public transportation. Household tasks are excluded to avoid gender-related biases.
5. Severe disability is defined as the number of basic activities of daily living (ADLs) that the person can not perform independently. ADLs, assessed using the Barthel Index, will include five essential self-care activities: bathing, toileting, transferring, and eating.

### Covariables

- *Short Falls Efficacy Scale International:* The Short Falls Efficacy Scale International (Short FES-I) is the abbreviated version of the FES-I, measuring fear of falling. It includes seven of the sixteen FES-I items, assessing how likely the participant believes they might fall in seven daily situations [42]. Responses range from 1 to 4: not at all concerned, somewhat concerned, fairly concerned, or very concerned. The total score ranges from 7 to 28, with higher scores indicating worse fear of falling [43].
- Record of falls and consequences during the year prior to study inclusion.
- Charlson Comorbidity Index, number of chronic conditions, and specific chronic comorbidities (ICD-10).
- Cognitive capacity assessed using the *Pfeiffer* short portable mental status questionnaire or *Mini Mental State Examination*.
- Standard eye acuity test.
- Subjective assessment of sense organs (visual/auditory): normal, mild impairment, or severe impairment.
- Need for technical assistance when walking (yes/no), and type of assistance used.
- Record of rehabilitation gait treatment in the past year.
- Loneliness: measurement of loneliness using the *UCLA-3 Loneliness Scale* and the *Jong Gierveld Loneliness Scale*.
- Blood sample for a future study of frailty/sarcopenia biomarkers. Aging is associated with increased proinflammatory cytokines. Elevated cytokines levels are also observed in sarcopenia, obesity, and osteoporosis (increased levels of IL6 and low levels of IGF-1), as well as other endocrine biomarkers (low dehydroepiandrosterone sulfate, vitamin D and leptin).
- Other variables: Affiliation data, numerical ID code, anthropometric measurements, regular drug treatment, socioeconomic status (education, financial level, civil status), and lifestyle factors (smoking habits, alcohol consumption.

### Retinography

Using a non-mydriatic camera, retinal images were obtained from each eye without pupillary dilation, capturing both central and nasal views. These images were then analyzed with the IMEDOS Vessel Map software (IMEDOS Systems UG, Germany), while the Tortuosity Index and A/V ratio were measured with the retinal analysis platform of the VARPA group from the University of A Coruña.

### Cohort follow-up

- Record of falls (indicating place and activity at the time) and consequences: contusions, fractures, hospitalization, or death within 30 days.
- Formal and informal social care (self-reported, hours/month).
- Hospital admissions, 30-day readmission, days spent in the hospital, primary care visits, and specialist care visits.

The same variables collected at baseline will be gathered again at the final follow-up visit.

### Data collection

A standardized data collection protocol and logbook will be established following a pilot test on 50 cases. All information and data collection will be conducted using an electronic form designed with RedCAP. This pilot test aims to detect design errors, train recruiters, and verify consistency in data collection to ensure a standardized process. A random sample of candidate subjects will be selected and contacted (up to six attempts on different days and times). With prior knowledge of their referring doctor or nurse, these candidates will be invited to participate in the study. An informative visit will be scheduled to review inclusion and exclusion criteria and to sign the informed consent form.

If included in the study, the complete baseline visit will be conducted at this time, except for the blood test, which will be scheduled within one month from inclusion. From this baseline visit, subjects will be followed up to detect falls by using: a) personal calendar for recording falls, and b) bimonthly phone calls.

**Fig 1:**
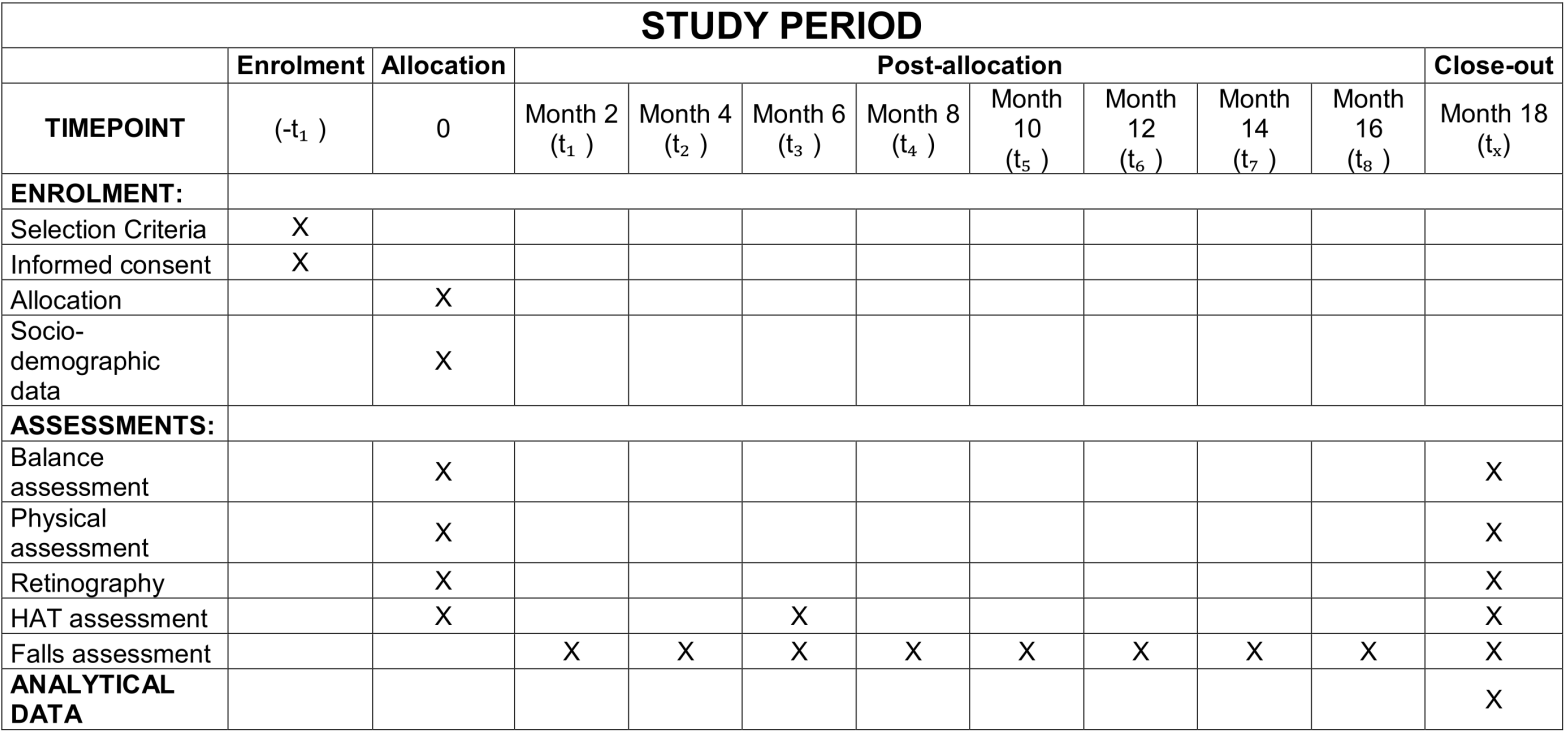
SPIRIT diagram illustrating the schedule for enrolment, interventions, and assessments.

**Fig 2:**
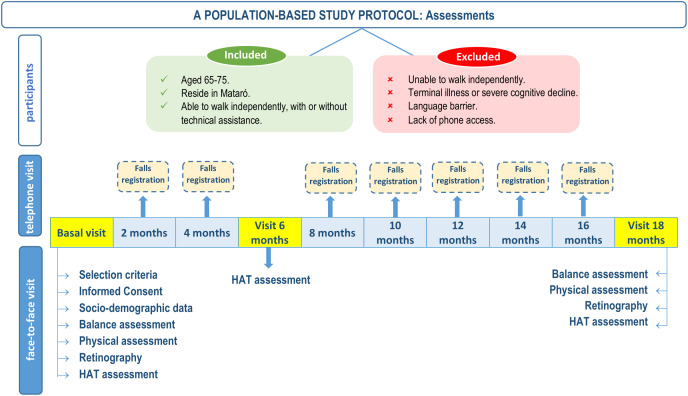
Flowchart of the study protocol and participant assessments.

The final visit will be conducted at 18 months, during which balance will be assessed again through clinical tests, retinography, and posturography. The detailed schedule for enrollment, interventions, and assessments is presented in the SPIRIT diagram (Fig 1). A flowchart illustrating the overall study protocol and participant flow is provided in Fig 2.

### Statistical analysis

Qualitative variables will be summarized as absolute and relative frequencies. Continuous variables will be described using mean and standard deviation or median and interquartile range, as appropriate. Distributional assumptions will be assessed using graphical methods, and the Shapiro-Wilk test will be used to check for data normality. Baseline characteristics between participants with and without balance disorders (BDs) will be compared using the Chi-square or Fisher’s exact test for categorical variables, and the Student’s t-test or Mann-Whitney U test for continuous variables, based on the distributional properties of the data.

BD prevalence and cumulative incidence at 18 months will be estimated with 95% confidence intervals (CIs). The association between BDs and the occurrence of at least one fall during the 18-month follow-up will be analyzed using a modified Poisson regression with robust variance to estimate relative risks (RRs) and 95% CIs. Additionally, a survival analysis for the time until the first fall will be performed using Kaplan-Meier survival curves and Cox regression.

To evaluate the predictive capacity of knee extensor strength and the composite physical-cognitive score for BDs and fall risk, separate multivariable modified Poisson regression models with robust variance will be constructed. Continuous predictors will be modeled as such, and adjusted RRs with 95% CIs will be reported. The diagnostic performance of the Wii Balance Board™ will be evaluated against posturography (gold standard) by calculating sensitivity, specificity, predictive values (PPV, NPV), likelihood ratios, and the area under the ROC curve (AUC). For the validation of the Health Assessment Tool (HAT), we will compare baseline values with those obtained at the 18-month follow-up. The predictive capacity of the HAT scale for BDs and health services utilization (e.g., hospital admissions, primary care visits) will be estimated by computing the AUC.

The analysis regarding retinal microvasculature (Aim 2) will be treated as an exploratory analysis. While we investigate the link between retinal vascular health and BDs using modified Poisson regression adjusted for predefined clinical confounders, this component primarily aims to identify potential new non-invasive biomarkers. Therefore, findings related to these parameters will be interpreted as hypothesis-generating for future research rather than primary confirmatory outcomes.

A two-sided p-value <0.05 will be considered statistically significant. All analyses will be performed using SPSS version 23.0.

### Risks and disadvantages for patients

There are minimal risks or disadvantages for the subjects arising from their participation in this study. The only anticipated disadvantages are the following: 1) Blood collection: the extraction of a blood sample for the future study of frailty and sarcopenia biomarkers may be accompanied by minor discomfort or temporary swelling or bruising at the puncture site. All standard clinical measures will be taken by trained health professionals to minimize these effects. 2) Retinal photography: While this involves taking images of the ocular fundus, the procedure is performed using a non-mydriatic camera. This method is quick, safe, and non-invasive, as it does not require pupillary dilation or the use of any medication. 3) Clinical and balance assessments: Procedures such as static posturography, knee extensor strength assessment, and balance tests using Wii Balance Board™ or standard clinical scales (Tinetti, TUG, SPPB) are completely non-invasive and do not involve radiation or painful maneuvers. 4) Follow-up: Some participants may experience a slight inconvenience due to the time required for the baseline and final visits, as well as the bimonthly phone calls used to track the occurrence of falls.

Overall, the benefits of participating in this study – including the early detection of balance disorders and the identification of potential fall risks – outweigh theses minimal risks.

### Ethical considerations

The study is being conducted in accordance with the Declaration of Helsinki, agreed at the 64th General Assembly in 2013. Confidentiality and anonymity of the data will be ensured in accordance with current state laws (Organic Law 3/2018, of 5 December, on Personal Data Protection and guarantee of digital rights), both during the execution phase and in any presentations or publications arising from the study. All data were anonymized and the confidentiality of Health Electronic Record (HER) was maintained at all times, in accordance with national and international law.

The research protocol has been reviewed and approved by the Ethics and Clinical Research Committee of the IDIAP Jordi Gol, Barcelona, Spain (protocol code P17/239, first date of approval 20/12/2017; date of approval of amendments 16/11/2022). For studies involving humans, it will be under its supervision during its development and will incorporate its recommendation. An informative sheet was distributed to the participants, and their informed consent was requested in writing.

Participants and their primary care doctors have access to the results of all clinical tests performed during the study through their electronic medical records. During the second visit, subjects are provided with their individual results from the baseline assessment. Upon completion of the study, the final report and any resulting publications will be sent to all participants via email or post to ensure they are informed of the study’s outcomes.

### Study status

The first participant in the study was enrolled in April 2022, and recruitment is expected to be completed by December 2025. Given that the data collection phase is still ongoing, no data analysis has been initiated, and therefore, the study has not yet generated any preliminary or final results.

## Discussion

Good balance is essential to prevent falls and promote individual autonomy. There is a documented link between balance and survival, where the successful execution of the one-legged stance for 10 seconds and the semi-tandem stance has been associated with increased survival rates [5,6]. However, the prevalence of BD in older individuals remains poorly understood due to the lack of consensus on evaluation methods.

Clinical tests like the Tinetti Test, despite its widespread use, have limitations such as the ceiling effect, making it less effective for assessing balance in community-dwelling older adults. Evaluating balance is complex as it indirectly assesses multiple systems, including the vestibular, visual, somatosensory, central nervous, and musculoskeletal systems. A comprehensive balance assessment is necessary to evaluate these systems together.

Static posturography offers an objective way to systematically understand postural control by providing sensitive information about the oscillations of the center of gravity in an upright static stance [44]. It is a fast, non-invasive, and effective tool for screening possible balance abnormalities [45]. However, the absence of a standardized protocol for quantitative posturography complicates comparisons between studies and limits its clinical applications.

To address these challenges, our study complemented static posturography with other established clinical tests, such as the Timed Up and Go Test, the Unipodal Test, the Short Physical Performance Battery (SPPB), and gait speed assessments. Our goal is to identify markers that best predict falls and provide a comprehensive balance assessment.

The primary objective of our study was to determine the prevalence and incidence of BD using posturography and other clinical tests. We aimed to enhance the epidemiological understanding of BD community-dwelling older adults and identify potential markers. Understanding the prevalence of these disorders is crucial for health professionals and policymakers to grasp the scope of the issue, thereby enabling more effective allocation of preventive resources.

Our findings support the need for a multifaceted approach in evaluating balance. For instance, though valuable, static posturography must be complemented by functional assessments to capture the full spectrum of balance capabilities and identify those at risk of falls. This approach aligns with the literature that emphasizes the multifactorial nature of balance and the necessity for diverse assessment tools [46,47].

It is essential to explore the integration of retinal microvasculature analysis into balance assessments. Previous research has demonstrated correlations between retinal abnormalities, cognition disorders, and cerebral ischaemia, suggesting that retinal imaging could serve as a non-invasive biomarker for identifying individuals with BD [48,49].

In conclusion, our study contributes to the growing body of knowledge on BD in older individuals. It advocates for a holistic assessment approach combining various clinical tests and innovative tools to effectively identify and address balance issues. Such comprehensive evaluations are imperative for developing targeted interventions to reduce falls and enhance the quality of life for older adults. Additionally, the study will help characterize and better understand the process of balance system deterioration associated with aging, contributing to the identification of non-invasive markers for early detection. This approach will be instrumental in the early identification of individuals at risk and in the development of more efficient preventive strategies.

### Limitations of the study

The study has several limitations. An estimated loss to follow-up of 10% per year is expected. Although the sample size has been calculated to compensate for this loss, efforts will be made to encourage continued participation through a communication line with the healthcare professionals responsible for each patient and by providing participants with their test results. There is also a risk of underreporting falls with minor consequences. To address this, a telephone interview protocol will be implemented to explore all types of falls. Additionally, variations in data collection may occur, but these will be minimized through thorough training of the study personnel. Furthermore, as the study protocol involves using a non-mydriatic camera without pupillary dilation, we anticipate challenges in acquiring high-quality retinal images from all participants. This may be particularly true in the presence of age-related factors like cataracts or small pupils, potentially reducing the number of samples available for analysis. Another potential limitation is a high non-response rate during the initial recruitment phase. To mitigate this, recruitment will be exhaustive and follow a strict protocol, including making calls on different days and at different times. In cases where incorrect phone numbers are encountered, referring healthcare providers will be contacted and informed. Furthermore, basic information on the reasons for non-participation will be collected to assess potential selection biases.

We acknowledge that some traditional clinical assessments, such as the Tinetti Test, may present a ceiling effect when applied to relatively healthy community-dwelling older adults. To mitigate this limitation and ensure the technical soundness of our protocol, we have established static posturography as our primary objective measure, as it provides quantitative data without floor or ceiling effects. Additionally, the Health Assessment Tool (HAT) has been included specifically because it lacks these limitations, allowing for a more sensitive evaluation of functional decline.

### Expected results and relevance

We’re addressing a prevalent health problem that has a clear social impact in the context of a progressively aging population. There is very little literature on the percentage of BD in the population, and a tendency not to report falls with minor consequences. This project is aimed at determining the magnitude of the problem so that it can later be addressed with greater knowledge. In this way, we will be able to respond to primary care doctors’ need for markers that make it possible to identify BD. By identifying subjects at the greatest risk of falling, we will be able to design more efficient preventive interventions (on the community and individual level).

The expected results of this study include a better understanding of the prevalence of BD in the population over 65 years old, which will allow us to extrapolate the percentage of affected individuals within this age group. We also expect to identify simple and accessible measures in primary care, such as gait speed or knee extensor strength, which can serve as indicators of BD. Furthermore, the study aims to determine whether retinography images are related to balance, contributing to the development of new diagnostic tools.

Moreover, the HAT index in our population would help the scientific community to have a simple health index that is a powerful predictor of disease and consumption of health services and resources. This validation could optimize the identification of individuals at greater risk, aiding in planning more effective public health programs.

Additionally, the findings are expected to raise awareness about BD, which are currently underdiagnosed and undertreated, through active dissemination in local media and social networks. Ultimately, these results could support the development of fall prevention and rehabilitation programs, helping to reduce the loss of autonomy in the elderly population.

## Conclusions

This study aims to provide new epidemiological knowledge on the incidence and prevalence of BD in older people and identify their possible markers for these disorders. Additionally, it seeks to validate the Health Assessment Tool (HAT) in the Spanish population. The expected results will provide a clearer understanding of the prevalence, incidence, and causes of BD, helping healthcare professionals tailor prevention and therapeutic programs to the specific needs of the elderly population.

## Supporting information

S1 File SPIRIT checklist. (DOCX)

S2 File Data protection and management. (DOCX)

S3 File Information sheet and consent for participants. (DOCX)
