# Supplementary material for: Prevalence and incidence of balance disorders in community-dwelling older adults: Protocol for the EPIBAS epidemiological balance study
Source: PLoS One. 2026 Jun 5;21(6):e0346698. doi: 10.1371/journal.pone.0346698 (PMC13240895; doi:10.1371/journal.pone.0346698)
Supplement: S1 File — (DOCX) [file pone.0346698.s001.docx]

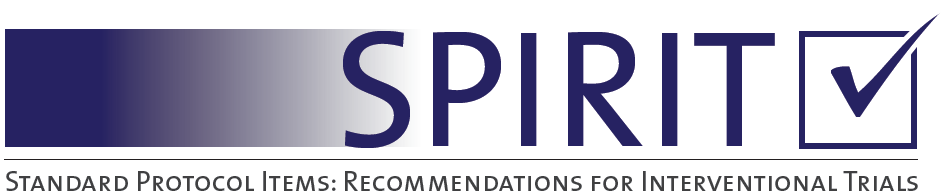


SPIRIT 2013 Checklist: Recommended items to address in a clinical trial protocol and related documents*

| Section/item | ItemNo | Description | Addressed on page number/heading |
| --- | --- | --- | --- |
| **Administrative information** | | |  |
| Title | 1 | Descriptive title identifying the study design, population, interventions, and, if applicable, trial acronym | Page 1 / "Prevalence and Incidence of Balance Disorders in Community-Dwelling Older Adults: Protocol for the EPIBAS Epidemiological Balance Study" |
| Trial registration | 2a | Trial identifier and registry name. If not yet registered, name of intended registry | Page 3 / Abstract – Registered at ClinicalTrials.gov (NCT06965660) |
|  | 2b | All items from the World Health Organization Trial Registration Data Set | Not applicable – information not included |
| Protocol version | 3 | Date and version identifier | Page 11 / Ethical considerations ("Protocol code P17/239") |
| Funding | 4 | Sources and types of financial, material, and other support | Page 14 / Funding Statement |
| Roles and responsibilities | 5a | Names, affiliations, and roles of protocol contributors | Page 13, 14 / Author Contributions |
|  | 5b | Name and contact information for the trial sponsor | Page 2 / Corresponding author |
|  | 5c | Role of study sponsor and funders, if any, in study design; collection, management, analysis, and interpretation of data; writing of the report; and the decision to submit the report for publication, including whether they will have ultimate authority over any of these activities | Page 14 / Funding Statement – “not involved in any way...” |
|  | 5d | Composition, roles, and responsibilities of the coordinating centre, steering committee, endpoint adjudication committee, data management team, and other individuals or groups overseeing the trial, if applicable (see Item 21a for data monitoring committee) | Page 13, 14 / Author Contributions |
| Introduction |  |  |  |
| Background and rationale | 6a | Description of research question and justification for undertaking the trial, including summary of relevant studies (published and unpublished) examining benefits and harms for each intervention | Page 4, 5 / Introduction |
|  | 6b | Explanation for choice of comparators | Page 5 / Comparison of the Wii Balance Board™ with posturography is warranted |
| Objectives | 7 | Specific objectives or hypotheses | Page 6 / Study Aims |
| Trial design | 8 | Description of trial design including type of trial (eg, parallel group, crossover, factorial, single group), allocation ratio, and framework (eg, superiority, equivalence, noninferiority, exploratory) | Page 6 / Methods – Two-phase, observational study in Epidemiology Criteria (STROBE) |
| Methods: Participants, interventions, and outcomes | | |  |
| Study setting | 9 | Description of study settings (eg, community clinic, academic hospital) and list of countries where data will be collected. Reference to where list of study sites can be obtained | Page 6 / Setting – Primary care centers in Mataró |
| Eligibility criteria | 10 | Inclusion and exclusion criteria for participants. If applicable, eligibility criteria for study centres and individuals who will perform the interventions (eg, surgeons, psychotherapists) | Page 6, 7 / Participants – Inclusion and exclusion criteria |
| Interventions | 11a | Interventions for each group with sufficient detail to allow replication, including how and when they will be administered | Not applicable – observational study |
|  | 11b | Criteria for discontinuing or modifying allocated interventions for a given trial participant (eg, drug dose change in response to harms, participant request, or improving/worsening disease) | Not applicable |
|  | 11c | Strategies to improve adherence to intervention protocols, and any procedures for monitoring adherence (eg, drug tablet return, laboratory tests) | Not applicable |
|  | 11d | Relevant concomitant care and interventions that are permitted or prohibited during the trial | Not applicable |
| Outcomes | 12 | Primary, secondary, and other outcomes, including the specific measurement variable (eg, systolic blood pressure), analysis metric (eg, change from baseline, final value, time to event), method of aggregation (eg, median, proportion), and time point for each outcome. Explanation of the clinical relevance of chosen efficacy and harm outcomes is strongly recommended | Page 6, 7/ Study Aims and Variables |
| Participant timeline | 13 | Time schedule of enrolment, interventions (including any run-ins and washouts), assessments, and visits for participants. A schematic diagram is highly recommended (see Figure) | Page 10 / Figure 1 - baseline visits, bimonthly phone calls and final visit at 18 months |
| Sample size | 14 | Estimated number of participants needed to achieve study objectives and how it was determined, including clinical and statistical assumptions supporting any sample size calculations | Page 7 / A detailed calculation of the sample size (1,316 subjects) is provided based on expected prevalence, incidence of falls, alpha and beta risk, and a dropout rate of 20% |
| Recruitment | 15 | Strategies for achieving adequate participant enrolment to reach target sample size | Page 6 / random selection from the SIAP database, telephone contact |
| **Methods: Assignment of interventions (for controlled trials)** | | |  |
| Allocation: |  |  |  |
| Sequence generation | 16a | Method of generating the allocation sequence (eg, computer-generated random numbers), and list of any factors for stratification. To reduce predictability of a random sequence, details of any planned restriction (eg, blocking) should be provided in a separate document that is unavailable to those who enrol participants or assign interventions | Not applicable – observational study |
| Allocation concealment mechanism | 16b | Mechanism of implementing the allocation sequence (eg, central telephone; sequentially numbered, opaque, sealed envelopes), describing any steps to conceal the sequence until interventions are assigned | Not applicable |
| Implementation | 16c | Who will generate the allocation sequence, who will enrol participants, and who will assign participants to interventions | Not applicable |
| Blinding (masking) | 17a | Who will be blinded after assignment to interventions (eg, trial participants, care providers, outcome assessors, data analysts), and how | Not applicable |
|  | 17b | If blinded, circumstances under which unblinding is permissible, and procedure for revealing a participant’s allocated intervention during the trial | Not applicable |
| **Methods: Data collection, management, and analysis** | | |  |
| Data collection methods | 18a | Plans for assessment and collection of outcome, baseline, and other trial data, including any related processes to promote data quality (eg, duplicate measurements, training of assessors) and a description of study instruments (eg, questionnaires, laboratory tests) along with their reliability and validity, if known. Reference to where data collection forms can be found, if not in the protocol | Page 10-14 / Data collection and assessments. All the instruments and variables to be collected are detailed. A standardized protocol, a pilot test and an electronic form in RedCAP will be used |
|  | 18b | Plans to promote participant retention and complete follow-up, including list of any outcome data to be collected for participants who discontinue or deviate from intervention protocols | Page 12 / Attempts will be made to minimize loss to follow-up through a line of communication and by providing participants with the results of their tests |
| Data management | 19 | Plans for data entry, coding, security, and storage, including any related processes to promote data quality (eg, double data entry; range checks for data values). Reference to where details of data management procedures can be found, if not in the protocol | Page 11 / Confidentiality and anonymity of data is guaranteed according to the Organic Law 3/2018. An electronic form (RedCAP) is used for data collection. |
| Statistical methods | 20a | Statistical methods for analysing primary and secondary outcomes. Reference to where other details of the statistical analysis plan can be found, if not in the protocol | Page 7 / Sample size.  The objectives of the analysis are described (e.g., to analyze association, validity, predictive capacity). The sample size calculation is based on the determination of a risk ratio of 1.3. However, a specific statistical analysis plan is not detailed. |
|  | 20b | Methods for any additional analyses (eg, subgroup and adjusted analyses) |  |
|  | 20c | Definition of analysis population relating to protocol non-adherence (eg, as randomised analysis), and any statistical methods to handle missing data (eg, multiple imputation) | Page 12 / Limitations – Dropout estimation and strategies |
| **Methods: Monitoring** | | |  |
| Data monitoring | 21a | Composition of data monitoring committee (DMC); summary of its role and reporting structure; statement of whether it is independent from the sponsor and competing interests; and reference to where further details about its charter can be found, if not in the protocol. Alternatively, an explanation of why a DMC is not needed | Page 11/ There is no mention of a formal DMC. However, it is indicated that the study will be under the supervision of the Clinical Research Ethics Committee during its development. |
|  | 21b | Description of any interim analyses and stopping guidelines, including who will have access to these interim results and make the final decision to terminate the trial |  |
| Harms | 22 | Plans for collecting, assessing, reporting, and managing solicited and spontaneously reported adverse events and other unintended effects of trial interventions or trial conduct | Page 6 / Falls and their consequences will be recorded by means of telephone calls |
| Auditing | 23 | Frequency and procedures for auditing trial conduct, if any, and whether the process will be independent from investigators and the sponsor | Not mentioned |
| Ethics and dissemination | | |  |
| Research ethics approval | 24 | Plans for seeking research ethics committee/institutional review board (REC/IRB) approval | Page 11 / Ethical considerations – Approval by CEIC IDIAP Jordi Gol |
| Protocol amendments | 25 | Plans for communicating important protocol modifications (eg, changes to eligibility criteria, outcomes, analyses) to relevant parties (eg, investigators, REC/IRBs, trial participants, trial registries, journals, regulators) | Page 11 / Ethical considerations – Amendments approved 16/11/2022 |
| Consent or assent | 26a | Who will obtain informed consent or assent from potential trial participants or authorised surrogates, and how (see Item 32) | Page 11 / Informed consent obtained in writing |
|  | 26b | Additional consent provisions for collection and use of participant data and biological specimens in ancillary studies, if applicable |  |
| Confidentiality | 27 | How personal information about potential and enrolled participants will be collected, shared, and maintained in order to protect confidentiality before, during, and after the trial | Page 11 / Confidentiality and anonymization measures |
| Declaration of interests | 28 | Financial and other competing interests for principal investigators for the overall trial and each study site | Page 19 / Conflict of Interest Statement |
| Access to data | 29 | Statement of who will have access to the final trial dataset, and disclosure of contractual agreements that limit such access for investigators | Page 11 / RedCAP data collection – data stored securely |
| Ancillary and post-trial care | 30 | Provisions, if any, for ancillary and post-trial care, and for compensation to those who suffer harm from trial participation | Not applicable |
| Dissemination policy | 31a | Plans for investigators and sponsor to communicate trial results to participants, healthcare professionals, the public, and other relevant groups (eg, via publication, reporting in results databases, or other data sharing arrangements), including any publication restrictions | Page 13 / The publication and presentation of the results is planned. There is a plan for active dissemination in local media and social networks. The author's contributions section details those responsible for the writing |
|  | 31b | Authorship eligibility guidelines and any intended use of professional writers |  |
|  | 31c | Plans, if any, for granting public access to the full protocol, participant-level dataset, and statistical code |  |
| Appendices |  |  |  |
| Informed consent materials | 32 | Model consent form and other related documentation given to participants and authorised surrogates | Page 13 / S2 File – Information sheet and consent |
| Biological specimens | 33 | Plans for collection, laboratory evaluation, and storage of biological specimens for genetic or molecular analysis in the current trial and for future use in ancillary studies, if applicable | Page 9 / Blood sample for biomarkers of frailty/sarcopenia |

*It is strongly recommended that this checklist be read in conjunction with the SPIRIT 2013 Explanation & Elaboration for important clarification on the items. Amendments to the protocol should be tracked and dated. The SPIRIT checklist is copyrighted by the SPIRIT Group under the Creative Commons “[Attribution-NonCommercial-NoDerivs 3.0 Unported](http://www.creativecommons.org/licenses/by-nc-nd/3.0/)” license.
