# Supplementary material for: Prevalence and incidence of balance disorders in community-dwelling older adults: Protocol for the EPIBAS epidemiological balance study
Source: PLoS One. 2026 Jun 5;21(6):e0346698. doi: 10.1371/journal.pone.0346698 (PMC13240895; doi:10.1371/journal.pone.0346698)
Supplement: S3 File — (DOCX) [file pone.0346698.s003.docx]

**
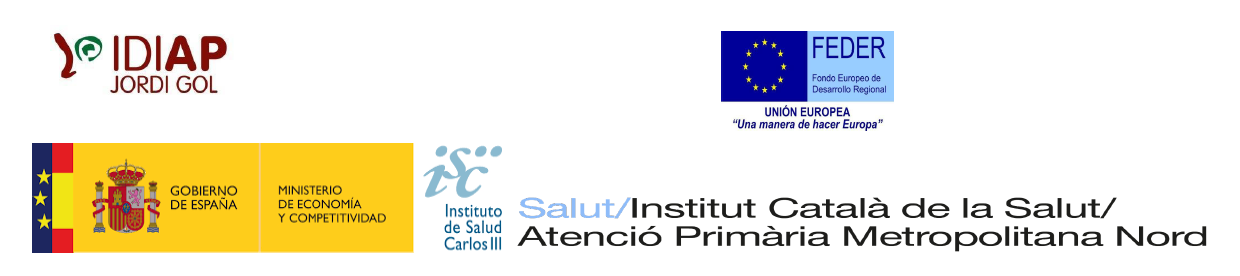
**

**PATIENT INFORMATION SHEET AND INFORMED CONSENT**

**Study Title:** ESTABLISHMENT OF A POPULATION COHORT FOR THE EPIDEMIOLOGICAL KNOWLEDGE OF BALANCE DISORDERS AND FALLS IN THE ELDERLY.

**1. INTRODUCTION:**

We are writing to you to provide information about a study in which you are invited to participate. This study has been approved by the Clinical Research Ethics Committee of the IDIAP Jordi Gol Institute.

Our intention is solely to ensure you receive correct and sufficient information to evaluate and decide whether or not to participate in this study. Please read this information sheet carefully, and we will clarify any doubts you may have after the explanation. Additionally, you may consult with anyone you deem appropriate.

**2. VOLUNTARY PARTICIPATION:**

You should know that your participation in this study is voluntary. You may decide not to participate and can withdraw your consent at any time without altering your relationship with your doctor/nurse or causing any prejudice to your treatment.

**3. GENERAL DESCRIPTION OF THE STUDY:**

The objectives of this study are, on one hand, to understand balance disorders by applying new balance markers: posturography, retinography, and quadriceps extensor strength. On the other hand, the study aims to validate the HAT (Health Assessment Tool) for identifying individuals at early risk of health deterioration and greater healthcare resource needs.

To carry out this validation, we will need to access data from your clinical history and consult socio-sanitary data. We will also perform a basic health blood draw.

This is a descriptive study that involves one in-person visit to assess various parameters related to mobility and balance. There will be telephone follow-ups every 2 months to inquire about falls, and a final visit at 18 months to repeat questionnaires, posturography, and retinography.

No medication will be administered during the study, nor will participants be required to perform any intervention different from their normal daily life. Approximately 1300 participants from the City of Mataró, aged 69 to 70, will be recruited. We kindly request maximum cooperation in recalling any falls and their consequences.

**4. BENEFITS AND RISKS OF PARTICIPATING IN THE STUDY:**

Falls represent a serious health problem due to their significant repercussions for the person who suffers them, their family, and healthcare institutions. Not everyone ages in the same way, and for this reason, we want to detect people whose health may be affected earlier than expected to identify those at risk and act preventively.

**5. CONFIDENTIALITY AND ANONYMITY:**

The entire research team is committed to conducting the data collection, analysis, and preparation of results confidentially and anonymously. In accordance with Regulation (EU) 2016/679 of the European Parliament and of the Council of 27 April 2016 on Data Protection and Organic Law 3/2018, of December 5, on the Protection of Personal Data and guarantee of digital rights, access to your personal information will be restricted to the research team and the Ethics Committee for Research (IDIAPJGol). They will be subject to the duty of secrecy inherent to their profession and will only access the data when necessary to verify the study's data and procedures, always maintaining its confidentiality.

**6. USE AND DISSEMINATION OF RESULTS:**

The information collected will be used in an aggregated form to evaluate the effect of the intervention and to improve it for future editions of the program. The results will be disseminated to different audiences and through various formats, including to the participants themselves (research reports, conferences, workshops, and publications in scientific journals).

**7. CONTACT PERSON FOR THE STUDY FOR ANY QUESTIONS AND RIGHT OF ACCESS, CANCELLATION, AND OPPOSITION OF DATA:**

For any questions about the project, or to exercise your right to modify your personal data, you can contact the study managers or the nursing staff with whom you sign the informed consent.

Dra. Pilar Montero Alía, Nurse: Carmina Rodríguez Pérez Tel. +34 93 7415338 Research Support Unit Project mobile: 634693435 (messages or WhatsApp only) e-mail: epibas@idiapjgol.info


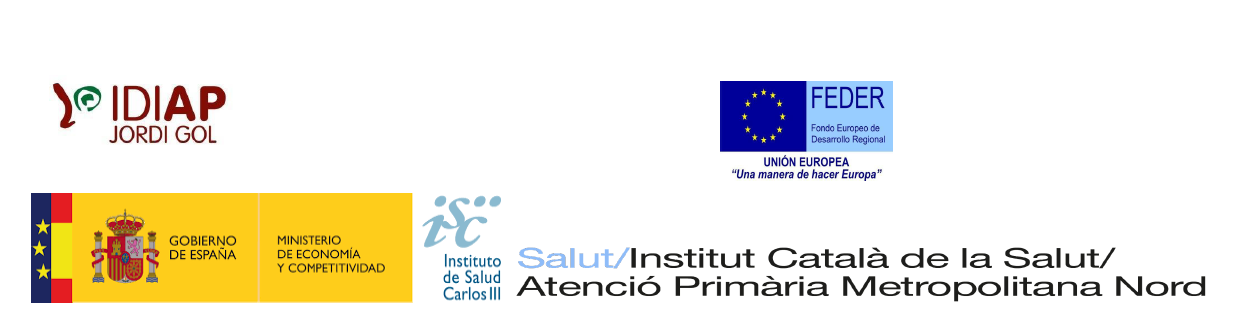
**Informed Consent**

**Study Title:** Establishment of a population cohort for the epidemiological knowledge of balance disorders and falls in the elderly.

I, ________________________________________________ (name and surname), with ID number ________________________

- I have read the participant information sheet that has been given to me.
- I have been able to ask questions about the study.
- I have sufficient information about the study.
- I grant permission to access my clinical history and consult my socio-sanitary situation.
- I grant permission for a blood draw and to store blood samples for future studies.
- I have spoken with ________________________________ (name and surname of the researcher).
- I understand what my participation in the study implies and that it is voluntary.
- I understand that I can withdraw from the study:
  1. Whenever I wish,
  2. Without giving explanations, and,
  3. Without any repercussions for me.

In accordance with the provisions of Regulation (EU) 2016/679 of the European Parliament and of the Council of April 27, 2016, on Data Protection and Organic Law 3/2018, of December 5, on the protection of personal data and guarantee of digital rights, I declare that I have been informed of my rights, the purpose of my participation in the study, the collection of my data, and the recipients of the information.

I freely give my consent to participate in this study.

Participant's name and surname and signature

Signature: Place and date:
